# Deafness and Additional Disabilities Questionnaire: translation and cultural adaptation into Brazilian Portuguese

**DOI:** 10.1590/2317-1782/e20240198en

**Published:** 2025-03-24

**Authors:** Vanessa Luisa Destro Fidêncio, Camila Rodrigues Cavalcante Arruda, Tatiane Franciele de Almeida, Anacleia Melo da Silva Hilgenberg

**Affiliations:** 1 Programa de Pós-graduação em Saúde da Comunicação Humana, Universidade Tuiuti do Paraná – UTP - Curitiba (PR), Brasil.; 2 CEAL-Ludovico Pavoni - Brasília (DF), Brasil.

**Keywords:** Surveys and Questionnaires, Translation, Cochlear Implantation, Child, Comorbidity

## Abstract

**Purpose:**

Translate and culturally adapt the Deafness and Additional Disabilities Questionnaire (DAD-Q) into Brazilian Portuguese.

**Methods:**

Translation, back-translation, review by an expert committee, and cultural adaptation were conducted. For the cultural adaptation, 11 mothers of deaf children with additional disabilities, who use cochlear implants, participated. The reliability of the translated instrument was estimated using internal consistency analysis (Cronbach's Alpha). Equivalence was defined as the absence of difficulty in understanding the questions by at least 80% of the participants. The participants also rated the difficulty in responding to the Brazilian Portuguese version of the instrument and the time required for its application.

**Results:**

The discrepancies in the translation were resolved by the expert committee, and 100% of the participants reported understanding all the questions in the instrument without difficulty. The Cronbach's alpha coefficient demonstrated nearly perfect internal consistency for the instrument and substantial consistency in the other domains. Most participants rated the instrument as "very easy" and "quick" to respond to.

**Conclusion:**

The DAD-Q instrument was translated and adapted into Brazilian Portuguese, resulting in the Questionário de Surdez e Deficiências Adicionais (DADQ-PT).

## INTRODUCTION

The cochlear implant (CI) revolutionized the treatment of severe or profound sensorineural hearing loss^(1)^, as it is a device that provides benefits for the development of hearing and communication skills in children when the procedure is held at an early age^(2)^. Apart from the age, other variables are involved in the obtained results with that hearing technology, including the surgical procedure and characteristics of the patient^(1)^. Evidence shows that comorbidities may influence the benefits obtained by the use of the cochlear implant among the pediatric population^(2)^, with a slower pattern of development of the communicative skills after CI in children with additional disabilities or impairments beyond the hearing loss, such as in cases with the presence of autism spectrum disorder (ASD)^(3)^, cerebral palsy (CP) and other developmental disorders^(4)^. In spite of that, there may be improvement in the hearing and language skills along the time, as well as benefits regarding the quality of life among that population^(5)^.

Assessing the development of infants can be challenging for professionals. Thus, the use of standardized questionnaires is common for monitoring auditory and language skills in children after the CI, applied in the form of interviews with parents^(6)^. There are several instruments in Brazilian Portuguese used to assess the progress of children who make use of the cochlear implant. However, they are usually standardized to be used with children without additional impairments. Considering that it is not rare for services to treat children with additional impairments to the hearing loss^(7,8)^, it is extremely important that the assessment covers this population as well.

In that sense, Palmieri et al.^(9)^ developed the instrument called Deafness and Additional Disabilities Questionnaire (DAD-Q). DAD-Q is a questionnaire which comprises 42 items, divided in five domains, elaborated to be applied in the form of an interview with parents/legal guardians in two situations: before and after the use of the cochlear implant. The authors classified the instrument as a simple, useful tool to assess the progress of children with hearing loss and additional disabilities, even in cases where progress is not detected by other tests routinely used in the clinical practice.

Therefore, the current study aims to translate and culturally adapt the DAD-Q questionnaire to Brazilian Portuguese.

## METHODS

It is a quantitative, prospective exploratory study with clinical design whose data collection was carried out at Ludovico Pavoni Educational Center of Hearing and Language - CEAL Ludovico Pavoni (CEAL-LP), in the city of Brasilia, Federal District. The study began after the approval of the Ethics Committee on Research of the Planalto Central University Center (Centro Universitário do Planalto Central - UNICEPLAC), opinion number 6.066.787. The participants signed the Free Informed Consent Formulary (Termo de Consentimento Livre e Esclarecido - TCLE).

DAD-Q comprises 47 items, divided in five domains, described below^(9)^:

Perceptual skills (DADQ-A): ten questions addressing the use of the device, perception and identification of environmental sounds and speech perception in quiet and noisy environments;
Preferred communication mode (DADQ-B): one question, which addresses the child’s communicative mode of preference, varying the communication only by means of his/her behavior to the use of full sentences;
Communicative behaviors (DADQ-C): nine questions addressing the child’s communicative behavior with family talkers and strangers regarding the communicative intent, communicative efficiency and vocal and gesture turn-taking;
Attention and memory skills (DADQ-D): eight questions about the focal and selective attention and long-term memory skills
Social interaction, behavior control, and self government (DADQ-E): 14 questions addressing the child’s behavior during interaction with family members and strangers, his/her control of emotional needs and independence during the activities of daily life.

In the DADQ-B domain, the score varies from 0 to 8 points, according to the child’s preferred mode of communication. To all the other four domains of the questionnaire, options of answers for each item include: “never” (0 points), “seldom” (1 point), “sometimes” (2 points), “often” (3 points), and “always” (4 points). Higher scores indicate better results in the abilities assessed in each domain and vice-versa.

At the end of the questionnaire, there are six additional questions, which must be applied only in the assessment after the cochlear implant, regarding the decision on the use of the device use. The answer options to those questions are “yes” or “no”, and they are not computed in the total score of the questionnaire.

The DAD-Q must be applied in the form of an interview, as the questions are designed to provoke a dialogue between the evaluator and the informant. The formulary filled in by parents voids the measure. Its objective is to characterize the benefit of the use of the cochlear implant in deaf children with additional disabilities^(9)^.

The translation of the DAD-Q into Brazilian Portuguese was authorized by the authors of the original instrument^(9)^ and followed the methodology proposed by Guillemin et al.^(10)^, including the steps described as follows:

Translation from English into Portuguese by two translators-interpreters of English, fluent in the language, who did not know each other and did not have previous knowledge of the questionnaire. This step resulted in two independent translations (T1 and T2);
Synthesis by an expert committee of two speech therapists, fluent in English, who carried out the analysis of the documents from the first step (T1 and T2), and reduced the differences found in the translations, generating a single document (T3);
Back translation from Portuguese into English by two translators-interpreters of English, different from the participants in the first step, who did not know the original text. The step resulted in two new documents (T4 and T5);
Review of the expert committee when the same professionals from the second step evaluated the versions T4 and T5., comparing them with the original version and generating again a single document (T6). Thus, a new and single questionnaire was obtained in Brazilian Portuguese (Appendix A);
Pre-test of equivalence (cultural adaptation), when the target population answered the instrument in Brazilian Portuguese as described below.

For the step of the pre-test equivalence, the following inclusion criteria were adopted: older than 18 years old; father or mother of a deaf child diagnosed with an additional disability and/or additional disabilities, and user of a cochlear implant. As exclusion criteria, the following criteria were adopted: parents of children who did not make use of the device in the six months prior to the date of the data collection; parents of children who did not have conclusive diagnosis of additional disabilities.

Eleven (11) mothers of deaf children with additional disabilities, and users of a cochlear implant, registered in the hearing rehabilitation service of the researched facility, who answered the translated version of the DAD-Q in the form of a face-to-face interview. All interviews were held by the same evaluator.

The reliability of the translated instrument was estimated by means of the internal consistency analysis (Cronbach’s Alpha), considering that α between 0 and 0.21 indicates low internal consistency, between 0.21 and 0.40 indicates reasonable internal consistency, between 0.41 and 0.60 indicates moderate internal consistency, between 0.61 and 0.80 indicates substantial internal consistency, and between 0.80 and 1.0 indicates almost perfect internal consistency^(11)^. The equivalence adopted was the absence of difficulty in understanding the questions by a minimum of 80% of the participants.

The participants also rated the difficulty in answering the Brazilian Portuguese version of the instrument (very difficult=0, difficult=1, moderate=2, easy=3 and very easy=4), and the length of time of application (very long=0, long=1, adequate=2, fast=3, very fast=4).

## RESULTS

The main divergences found in the process of translation are in Chart 1. There were no changes in the meaning of the expressions after the consensus.

**Chart 1:** Main discussions on the definition of the consensus

	Original version item in Englishs^(8)^	Translation into Brazilian Portuguese	Expert committee	Final version translated and culturally adapted
**Application guidelines**	*Administration*	T1 – Administração	Aplicação	Aplicação
		T2 - Aplicação		
	*Interviewer*	T1 – Entrevistador	Examinador	Examinador
		T2 - Entrevistador		
**Item 01 (Domain A)**	*Your child*	T1 - Seu filho	A criança	A criança
		T2 – Seu filho		
**Item 02 (Domain C)**	*Tries*	T1 - Dispõe-se	Tenta	Tenta
		T2 – Tenta		
**Item 05 (Domain D)**	*Interference stimuli*	T1 – Estímulos de interferência	Outros estímulos	Outros estímulos
		T2 – Outros estímulos		
**Item 01 (Domain E)**	*Hails*	T1 – Saúda	Acena	Acena
		T2 - Acena		
**Item 02 (Domain E)**	*Has appropriate interaction*	T1 – Tem interação	Interage de maneira apropriada	Interage de maneira apropriada
		T2 - Interage		
**Item 07 (Domain E)**	*Washing themself*	T1 - Lavar-se	Tomar banho	Tomar banho
		T2 - Lavar-se		
	*Having lunch/dinner*	T1 - Almoçar/jantar	Comer	Comer
		T2 - Almoçar/ jantar		
	*Early age*	T1 – Pouca idade	Criança muito pequena	Criança muito pequena
		T2 - Criança jovem		
	*Motor problems*	T1 - Problemas motores	Alterações motoras	Alterações motoras
		T2 – Problemas motores		
**Item 14 (Domain E)**	*Loves*	T1 – Adora	Gosta	Gosta
		T2 - Adora		
**Additional question No. 05**	*Multiple impairments*	T1 – Múltiplas deficiências	Deficiências adicionais	Deficiências adicionais
		T2 – Deficiências adicionais		
**Additional question No. 06**	*If you had*	T1 - Tivesse que	Precisasse	Precisasse
		T2 - Precisasse		

**Caption:** T1=Translator 1; T2=Translator 2

The title of the questionnaire after the translation into Brazilian Portuguese was defined as “*Questionário de Surdez e Deficiências Adicionais*” (DADQ-PT)”. All participants (n=11) reported that they could understand the questions of the instrument without any difficulties, showing the equivalence between the original instrument and the translated version.

Almost perfect internal consistency of the DADQ-PT instrument was obtained and substantial consistency in all the other domains (Table 1). The coefficient was not calculated separately for the domain DADQ-B (preferred communication mode) as it comprised a single question.

**Table 1:** Cronbach’s alpha coefficient values for the domains of DADQ-PT

Domain	Alfa Cronbach
Perceptual skills	0.85
Communicative Behaviors	0.72
Attention and memory	0.73
Social interaction. behavior control and self government	0.68
Total	0.91

Regarding participants’ educational level, 36.37% (n=4) had Higher Education, 27.28% (n=3) had Higher School, 27.28% (n=3) incomplete Higher School, and 9.09% (n=1) incomplete Middle School. Considering the easiness to answer the DADQ-PT, 72.73% (n=8) considered the instrument “very easy”, 9.09% (n=1) as “easy”, and 18.18% (n=2) as “moderate”. Regarding the time length to answer it, 27.28% (n=3) considered “very fast”, 54.54% (n=6) as “fast”, and 18.18% (n=2) as “adequate”.

## DISCUSSION

Children with congenital deafness, who had the cochlear device implanted before 12 months of chronological age, present potential to develop receptive and expressive language skills similarly to their listening peers^(12)^. In this sense, it is necessary to assess younger children, and many times it is not possible the direct assessment of their abilities, thus, it is common the use of questionnaires responded by their parents.

In Brazil, in order to assess implanted infants, the versions in Brazilian Portuguese of the Infant Toddler Meaningful Auditory Integration Scale (IT-MAIS)^(13)^, the LittlEars® Auditory Questionnaire^(14)^, and Meaningful Use of Speech Scale (MUSS)^(15)^, among others, are commonly used. The first two questionnaires assess the auditory perception of speech, while MUSS assesses the use of spoken language. However, these instruments were standardized for children without additional disabilities.

Assuming that deaf children with multiple disabilities commonly have a slower communicative development after the cochlear implant^(2-4)^, the elaboration of instruments aiming at that population is fundamental.

To this date, there are no studies conducted in Brazil with the use of the DAD-Q, as the instrument was only available in the English version^9^. The translation and cultural adaptation of the questionnaire aimed to fill a gap in the assessment of the cochlear implant benefit in deaf children with additional disabilities. The questionnaire not only assesses the auditory perception and spoken language, but also considers the child’s communicative performance, including questions on attention, interaction and preferred communication mode. It is important to assess children with additional disabilities, who, many times, may not present the expected benefit of the cochlear implant in their oral communication. However, they may show that in other communicative abilities, such as in the interaction^(16)^. Moreover, considering essential that the professional advises parents on their expectations about the use of the cochlear implant^(5)^, the application of the DADQ-PT for the assessment of deaf children with diverse additional disabilities may help provide information on what to expect with the use of the device in those populations, contributing to guide professional advice.

The validation of instruments after translation is a continuous process. Thus, further research is suggested by means of the use of the DADQ-PT in larger and varied samples for its standardization in the country.

## CONCLUSION

The Deafness and Additional Disabilities Questionnaire (DAD-Q) was translated and culturally adapted to Brazilian Portuguese, becoming the “*Questionário de Surdez e Deficiências Adicionais*” (DADQ-PT), making available an original and useful instrument for professionals in the area of auditory rehabilitation to assess the benefit of the cochlear implant in children with hearing loss and additional disabilities.

## Appendix A Questionário de Surdez e Deficiências Adicionais (DADQ-PT)

**Table:**

**Questionário de Surdez e Deficiências Adicionais (DADQ-PT)**									
**Avaliação do desenvolvimento comportamental, auditivo, comunicativo, atencional e social, pré e pós-implante coclear**									
**PESQUISA PARENTAL**									
**Nome__________________________ Preenchido por______________________ Data____________**									
**□Pré-IC □ Pós-IC**									
**Aplicação:** este questionário deve ser aplicado em formato de entrevista. As perguntas foram elaboradas para elucidar um diálogo entre examinador e informante. Fazer com que os próprios informantes (ex.: pais) preencham o instrumento, invalida o procedimento.									
**Instruções para o examinador:** pergunte ao informante: “Gostaríamos de saber sobre suas impressões a respeito do desenvolvimento comportamental, auditivo e comunicativo de seu filho. A cada pergunta, tente se lembrar e me contar o máximo de exemplos possíveis sobre esse comportamento e depois tente definir com que frequência a criança demonstra esse comportamento específico, de forma sistemática.”									
**Resposta**			**Abreviatura**	**Escore**
**Nunca**			N	0
**Raramente**			R	1
**Algumas vezes**			AV	2
**Muitas Vezes**			MV	3
**Sempre**			S	4
**A) Habilidades Perceptivas**										
1		Por quanto tempo a criança usa o implante coclear durante as horas em que está acordado?						N R AV MV S		
		(N: [0%]; R: [1%-25%]; AV [25%-50%]; MV [50%-75%]; S [>75%])								
2		Liga o processador de fala sozinho ou pede a alguém que o faça (ex.: aponta para o dispositivo, mesmo sem verbalização)?						N R AV MV S		
3		Percebe quando o dispositivo não está funcionando adequadamente (ex.: qualidade/volume)?						N R AV MV S		
4		Atende ao próprio nome quando chamado inesperadamente em um local silencioso (sem nenhuma pista visual)?						N R AV MV S		
5		Atende ao próprio nome quando chamado em um local ruidoso?						N R AV MV S		
6		Detecta o barulho de eletrodomésticos?						N R AV MV S		
7		Reage a sons em ambiente domiciliar (telefone tocando, batidas à porta, campainha etc.)?						N R AV MV S		
8		Reage aos sons de animais (miado, latido etc.)?						N R AV MV S		
9		Reconhece um animal pelo som que ele faz (ex: associa latido a um cachorro)?						N R AV MV S		
10		Percebe quando alguém está falando mesmo sem estar vendo a pessoa?						N R AV MV S		
**B) Modo preferencial de comunicação**										
Qual é o modo preferido utilizado pela criança para se comunicar?										
(0) Comportamento (ex. sorrisos, gritos, expressões faciais)										
(1) Gestos										
(2) Gestos e vocalizações										
(3) LIBRAS ou CSA (Comunicação Suplementar e Alternativa)										
(4) Gestos associados a sílabas										
(5) Palavras associadas a gestos										
(6) Palavras isoladas										
(7) Frases simples (2-3 palavras)										
(8) Sentenças completas										
**C) Comportamentos comunicativos**									
1		Tenta se comunicar com conhecidos, ex.: familiares, amigos, professores etc. (utilizando qualquer modo de comunicação)?				N R AV MV S			
2		Tenta se comunicar com desconhecidos (utilizando qualquer modo de comunicação)?				N R AV MV S			
3		Respeita as trocas de turnos na conversação?				N R AV MV S			
4		Sua fala é inteligível para seus familiares?				N R AV MV S			
5		Sua fala é inteligível para desconhecidos?				N R AV MV S			
6		Tenta se corrigir se as pessoas não o(a) entendem (utilizando qualquer modo de comunicação)?				N R AV MV S			
7		Consegue se comunicar ao telefone?				N R AV MV S			
8		Utiliza a fala como o modo de comunicação preferido para chamar alguém?				N R AV MV S			
9		Consegue comunicar suas necessidades (utilizando qualquer modo de comunicação)?				N R AV MV S			
**D) Atenção e habilidades de memória**									
1		É capaz de focar a atenção no ambiente?					N R AV MV S		
2		É capaz de focar a atenção em uma tarefa por tempo suficiente para executá-la?					N R AV MV S		
3		Demonstra escutar o que está sendo dito ou comunicado a ele(a)?					N R AV MV S		
4		Evita comportamentos como correr e subir nos lugares ou contorcer-se em sua cadeira ou remexer suas mãos ou pés em momentos inadequados?					N R AV MV S		
5		É capaz de manter sua atenção em uma tarefa e realizá-la, mesmo na presença de outros estímulos?					N R AV MV S		
6		Parece se lembrar de pessoas conhecidas (ex: parentes, professores, terapeutas)?					N R AV MV S		
7		É capaz de se lembrar de palavras conhecidas?					N R AV MV S		
8		É capaz de se lembrar das regras de um jogo simples?					N R AV MV S		
**E) Interação social, controle comportamental e autorregulação**									
1		Acena espontaneamente ao encontrar pessoas conhecidas?					N R AV MV S		
2		Interage adequadamente com outros (chama sua atenção; mantém distância recíproca; respeita os turnos da conversação)					N R AV MV S		
3		Gosta de ir à escola?					N R AV MV S		
4		Interage com familiares de maneira apropriada?					N R AV MV S		
5		Interage com os colegas de classe de maneira apropriada?					N R AV MV S		
6		Gosta de fazer as sessões de reabilitação?					N R AV MV S		
7		Age de forma independente durante as atividades de vida diária (ex.: tomar banho, comer etc.)? No caso de criança muito pequena ou com alterações motoras: parece desejar agir de forma independente?					N R AV MV S		
8		Demonstra ampla gama de emoções e afetos (ex.: feliz ao brincar com os colegas, concentrado ao fazer exercícios, triste ao ser castigado?)					N R AV MV S		
9		Fica feliz por estar na companhia de alguém?					N R AV MV S		
10		Entende piadas?					N R AV MV S		
11		Inicia uma atividade espontaneamente (ex.: escolhe e propõe jogos por conta própria)?					N R AV MV S		
12		Compreende quando é repreendido?					N R AV MV S		
13		Gosta de assistir TV?					N R AV MV S		
14		Gosta de escutar música?					N R AV MV S		
**Perguntas adicionais (somente para avaliação pós-IC)**									
1		Você acha que recebeu informações suficientes do centro de implante coclear na época para tomar a decisão correta?					SIM NÃO		
2		Você conheceu outras famílias de crianças implantadas com outras deficiências adicionais antes do implante de seu(sua) filho (a), cuja experiência o(a) ajudou a tomar sua decisão?					SIM NÃO		
3		Você sugeriria o implante coclear para um outro pai que tenha um filho com condição semelhante?					SIM NÃO		
4		Sua família concordou com a sua decisão?					SIM NÃO		
5		A criança foi diagnosticada com deficiências adicionais antes do implante coclear?					SIM NÃO		
6		Se você precisasse tomar a decisão sobre um implante coclear para seu (sua) filho(a) novamente, você optaria pelo implante coclear?					SIM NÃO		
**Escores**								
**A**	**Habilidades perceptivas**							
**B**	**Modo preferencial de comunicação**							
**C**	**Comportamentos comunicativos**							
**D**	**Atenção e habilidades de memória**							
**E**	**Interação social, controle comportamental e autorregulação**							
**Escore total**								
