# Supplementary material for: Deafness and Additional Disabilities Questionnaire: translation and cultural adaptation into Brazilian Portuguese
Source: Codas. 2025 Mar 24;37(3):e20240198. doi: 10.1590/2317-1782/e20240198en (PMC11963883; doi:10.1590/2317-1782/e20240198en)
Supplement: Translation

# Translation: Deafness and Additional Disabilities Questionnaire: tradução e adaptação cultural para o português brasileiro

**DOI:** 10.1590/2317-1782/e20240198pt

**Authors:** Vanessa Luisa Destro Fidêncio, Camila Rodrigues Cavalcante Arruda, Tatiane Franciele de Almeida, Anacleia Melo da Silva Hilgenberg

**Affiliations:** 1 Programa de Pós-graduação em Saúde da Comunicação Humana, Universidade Tuiuti do Paraná – UTP - Curitiba (PR), Brasil.; 2 CEAL-Ludovico Pavoni - Brasília (DF), Brasil.

**Keywords:** Inquéritos e Questionários, Tradução, Implante Coclear, Criança, Comorbidade

## Abstract

**Objetivo:**

Traduzir e adaptar culturalmente o instrumento Deafness and Additional Disabilities Questionnaire (DAD-Q) para o Português Brasileiro.

**Método:**

Realizou-se a tradução, retrotradução, revisão por comitê de especialistas e adaptação cultural. Para a adaptação cultural, participaram 11 mães de crianças surdas com deficiências adicionais, usuárias de implante coclear. A confiabilidade do instrumento traduzido foi estimada utilizando-se a análise de consistência interna (Alfa de Cronbach). Adotou-se como equivalência a ausência de dificuldade de compreensão das questões por, no mínimo, 80% das participantes. As participantes também classificaram a dificuldade em responder à versão no português brasileiro do instrumento e o tempo de aplicação.

**Resultados:**

As divergências na tradução foram resolvidas pelo comitê de especialistas e 100% das participantes afirmaram compreender todas as questões do instrumento, sem dificuldades. O coeficiente alfa de Cronbach demonstrou consistência interna quase perfeita do instrumento e substancial nos demais domínios. A maioria das participantes classificou o instrumento como “muito fácil” e “rápido” de responder.

**Conclusão:**

O instrumento DAD-Q foi traduzido e adaptado para o Português Brasileiro, resultando no “Questionário de Surdez e Deficiências Adicionais (DADQ-PT)”.

## INTRODUÇÃO

O implante coclear (IC) revolucionou o tratamento da perda auditiva sensorioneural de grau severo ou profundo^(1)^, sendo um dispositivo que proporciona benefícios para o desenvolvimento das habilidades auditivas e comunicação em crianças quando realizado precocemente^(2)^. Além da idade da realização da cirurgia, outras variáveis estão envolvidas nos resultados obtidos com essa tecnologia auditiva, incluindo o procedimento cirúrgico e características do próprio paciente^(1)^. Evidências demonstram que comorbidades podem influenciar nos benefícios obtidos com o uso do implante coclear na população pediátrica^(2)^, havendo um padrão mais lento do desenvolvimento das habilidades comunicativas pós-IC em crianças com deficiências ou comprometimentos adicionais além da perda auditiva, como nos casos de presença de transtorno do espectro autista (TEA)^(3)^, paralisia cerebral (PC) e outros distúrbios do desenvolvimento^(4)^. Apesar disso, pode haver melhora das habilidades auditivas e de linguagem ao longo do tempo e benefícios no que diz respeito à qualidade de vida nessa população^(5)^.

Avaliar o desenvolvimento de crianças pequenas constitui um desafio para o profissional e, no monitoramento das habilidades auditivas e de linguagem de crianças após implantação, é comum o uso de questionários padronizados, aplicados em formato de entrevista com os pais^(6)^. Existem diversos instrumentos no português brasileiro utilizados para avaliar o progresso da criança com o uso do implante coclear. No entanto, estes costumam ser padronizados para o uso com crianças sem deficiências adicionais. Considerando que não é raro que os serviços recebam crianças que apresentam deficiências adicionais à deficiência auditiva^(7,8)^, torna-se extremamente necessária a realização de avaliação direcionada para essa população.

Nesse sentido, Palmieri et al.^(9)^ desenvolveram o instrumento *Deafness and Additional Disabilities Questionnaire* (DAD-Q). O DAD-Q é um questionário composto por 42 itens, divididos em cinco domínios, que foi elaborado no intuito de ser aplicado em formato de entrevista com os pais/responsáveis, em duas situações: antes e após o uso do implante coclear. Os autores classificaram o questionário como uma ferramenta simples e útil para avaliar o progresso de crianças com perda auditiva que apresentem deficiências adicionais, mesmo nos casos em que tais progressos não são detectados por outros exames rotineiramente utilizados na prática clínica.

Diante do exposto, o objetivo do presente estudo foi traduzir e adaptar culturalmente o instrumento DAD-Q para o português brasileiro.

## MÉTODO

Trata-se de um estudo de desenho clínico de caráter quantitativo, exploratório prospectivo, cuja coleta de dados foi realizada no CEAL-Ludovico Pavoni (CEAL-LP), na cidade de Brasília, Distrito Federal. O estudo teve início após aprovação do Comitê de Ética em Pesquisas do Centro Universitário do Planalto Central (UNICEPLAC), sob parecer nº 6.066.787. Os participantes assinaram o Termo de Consentimento Livre e Esclarecido (TCLE).

O DAD-Q é composto por 47 itens, divididos em cinco domínios, descritos abaixo^(9)^:

Habilidades perceptivas (DADQ-A): dez questões que abordam o uso do dispositivo, percepção e identificação de sons ambientais e percepção da fala em ambientes silenciosos e ruidosos;
Modo preferencial de comunicação (DADQ-B): uma questão, que aborda o modo comunicativo de preferência da criança, que varia de comunicação somente por meio do comportamento ao uso de frases completas;
Comportamentos comunicativos (DADQ-C): nove questões que abordam o comportamento comunicativo da criança com familiares e desconhecidos, em relação à intenção comunicativa, eficiência comunicativa e troca de turnos vocais e gestuais;
Atenção e memória (DADQ-D): oito questões direcionadas à atenção focalizada e seletiva e às habilidades de memória de longo prazo;
Interação social, controle do comportamento e autorregulação (DADQ-E): 14 questões abordam o comportamento da criança durante a interação com familiares e estranhos, seu controle quanto às suas necessidades emocionais e sua independência durante as atividades cotidianas.

No domínio DADQ-B, a pontuação varia de 0 a 8 pontos, de acordo com o modo de comunicação preferencial da criança. Para todos os outros quatro domínios do questionário, as opções de resposta de cada item incluem: “nunca” (0 pontos), “raramente” (1 ponto), “algumas vezes” (2 pontos), “muitas vezes “ (3 pontos) e “sempre” (4 pontos). Pontuações maiores indicam melhores resultados das habilidades avaliadas em cada domínio e vice-versa.

Ao final do questionário, constam seis questões adicionais, que devem ser aplicadas somente na avaliação após o implante coclear e dizem respeito à decisão sobre o uso do dispositivo. As opções de resposta para essas questões são “sim” ou “não” e não computam na pontuação total do questionário.

O DAD-Q deve ser aplicado em formato de entrevista, visto que as perguntas são projetadas para provocarem um diálogo entre o avaliador e o informante. O formulário preenchido diretamente pelos pais invalida a medida. Seu objetivo é caracterizar o benefício do uso do implante coclear por crianças surdas com deficiências adicionais^(9)^.

A tradução do DAD-Q para o português brasileiro foi autorizada pelos autores do instrumento original^(9)^ e seguiu a metodologia proposta por Guillemin et al.^(10)^, incluindo as etapas descritas a seguir:

Tradução do inglês para o português por dois tradutores-intérpretes de inglês, fluentes no idioma, que não se conheciam e não apresentavam conhecimento prévio do questionário. A etapa resultou em duas traduções independentes (T1 e T2);
Síntese pelo comitê de especialistas, formado por dois profissionais fonoaudiólogos fluentes na língua inglesa, que realizaram a análise dos documentos resultantes da primeira etapa (T1 e T2) e reduziram as diferenças encontradas nas traduções, gerando um único documento (T3);
Retrotradução do português para o inglês por dois tradutores-intérpretes de inglês, diferentes dos participantes da primeira etapa e desconhecedores do texto original. A etapa resultou em dois novos documentos (T4 e T5);
Revisão pelo comitê de especialistas, quando os mesmos profissionais da segunda etapa avaliaram as versões T4 e T5, comparando-as com a versão original em inglês e gerando, novamente, um único documento (T6). Assim, foi obtido um novo e único questionário no Português Brasileiro (Apêndice A);
Pré-teste de equivalência (adaptação cultural), quando a população alvo respondeu ao instrumento no Português Brasileiro, conforme descrito abaixo.

Para a etapa de pré-teste de equivalência, foram adotados os critérios de inclusão: ser maior de 18 anos; pai ou mãe de criança surda, diagnosticada com deficiência(s) adicional(is) e usuária de implante coclear. Foram adotados como critérios de exclusão: pais de crianças que não estivessem fazendo uso do dispositivo nos seis meses que antecederam a data da coleta de dados; pais de crianças que não apresentassem diagnóstico concluído de deficiências adicionais.

Participaram 11 mães de crianças surdas com deficiências adicionais, usuárias de implante coclear, matriculadas no serviço de (re)habilitação auditiva do local de pesquisa, que responderam presencialmente à versão traduzida do DAD-Q, no formato de entrevista. Todas as entrevistas foram realizadas pela mesma avaliadora.

A confiabilidade do instrumento traduzido foi estimada utilizando-se a análise de consistência interna (Alfa de *Cronbach*), considerando que α entre 0 a 0,21 indica pequena consistência interna, entre 0,21 e 0,40 indica consistência interna razoável, entre 0,41 e 0,60 indica consistência interna moderada, entre 0,61 a 0,80 indica consistência interna substancial e entre 0,80 e 1,0 indica consistência interna quase perfeita^(11)^. Adotou-se como equivalência a ausência de dificuldade de compreensão das questões por, no mínimo, 80% dos participantes.

Os participantes também classificaram a dificuldade em responder à versão no português brasileiro do instrumento (muito difícil=0, difícil=1, moderado=2, fácil=3 e muito fácil=4) e o tempo de aplicação (muito demorado=0, demorado=1, adequado=2, rápido=3, muito rápido=4).

## RESULTADOS

As principais divergências encontradas no processo de tradução estão no Quadro 1. Ressalta-se que não houve alteração no significado das expressões após o consenso.

**Quadro 1:** Principais discussões para a definição do consenso

	Item da versão original em inglês^(8)^	Tradução para o português brasileiro	Comitê de especialistas	Versão final traduzida e culturalmente adaptada
**Orientações de aplicação**	*Administration*	T1 – Administração	Aplicação	Aplicação
		T2 - Aplicação		
	*Interviewer*	T1 – Entrevistador	Examinador	Examinador
		T2 - Entrevistador		
**Item 01 (Domínio A)**	*Your child*	T1 - Seu filho	A criança	A criança
		T2 – Seu filho		
**Item 02 (Domínio C)**	*Tries*	T1 - Dispõe-se	Tenta	Tenta
		T2 – Tenta		
**Item 05 (Domínio D)**	*Interference stimuli*	T1 – Estímulos de interferência	Outros estímulos	Outros estímulos
		T2 – Outros estímulos		
**Item 01 (Domínio E)**	*Hails*	T1 – Saúda	Acena	Acena
		T2 - Acena		
**Item 02 (Domínio E)**	*Has appropriate interaction*	T1 – Tem interação	Interage de maneira apropriada	Interage de maneira apropriada
		T2 - Interage		
**Item 07 (Domínio E)**	*Washing themself*	T1 - Lavar-se	Tomar banho	Tomar banho
		T2 - Lavar-se		
	*Having lunch/dinner*	T1 - Almoçar/jantar	Comer	Comer
		T2 - Almoçar/ jantar		
	*Early age*	T1 – Pouca idade	Criança muito pequena	Criança muito pequena
		T2 - Criança jovem		
	*Motor problems*	T1 - Problemas motores	Alterações motoras	Alterações motoras
		T2 – Problemas motores		
**Item 14 (Domínio E)**	*Loves*	T1 – Adora	Gosta	Gosta
		T2 - Adora		
**Questão adicional nº 05**	*Multiple impairments*	T1 – Múltiplas deficiências	Deficiências adicionais	Deficiências adicionais
		T2 – Deficiências adicionais		
**Questão adicional nº 06**	*If you had*	T1 - Tivesse que	Precisasse	Precisasse
		T2 - Precisasse		

**Legenda:** T1=Tradutor 1; T2=Tradutor 2

O título do questionário após a tradução para o Português Brasileiro foi definido como “Questionário de Surdez e Deficiências Adicionais (DADQ-PT)”. Todas os participantes (n=11) afirmaram compreender todas as questões do instrumento, sem dificuldades, demonstrando a equivalência entre o instrumento original e a versão traduzida.

Obteve-se consistência interna quase perfeita do instrumento DADQ-PT e substancial nos demais domínios (Tabela 1). O coeficiente não foi calculado separadamente para o domínio DADQ-B (modo preferencial de comunicação), por tratar-se de uma única questão.

**Tabela 1:** Valores do coeficiente de alfa de Cronbach para os domínios do questionário DADQ-PT

Domínio	Alfa de Cronbach
Habilidades perceptivas	0,85
Comportamentos comunicativos	0,72
Atenção e memória	0,73
Interação social, controle do comportamento e autorregulação	0,68
Total	0,91

Com relação à escolaridade das participantes, 36,37% (n=4) apresentavam Ensino Superior completo, 27,28% (n=3) apresentavam Ensino Médio completo, 27,28% (n=3) Ensino Médio incompleto e 9,09% (n=1) Ensino Fundamental incompleto. Com relação à facilidade para responder ao DADQ-PT, 72,73% (n=8) classificaram o instrumento como “muito fácil”, 9,09% (n=1) como “fácil” 18,18% (n=2) como “moderado”. Com relação ao tempo para responder, 27,28% (n=3) classificaram como “muito rápido”, 54,54% (n=6) como “rápido” e 18,18% (n=2) como “adequado”.

## DISCUSSÃO

Crianças com surdez congênita que recebem o implante coclear antes dos 12 meses de idade cronológica, apresentam potencial para desenvolver as habilidades de linguagem receptiva e expressiva de maneira equiparada aos seus pares ouvintes^(12)^. Nesse sentido, têm-se a necessidade de avaliar crianças cada vez mais jovens, muitas vezes não sendo possível a avaliação direta das habilidades, sendo comum o uso de questionários realizados com os pais.

No Brasil, são comumente utilizados para avaliação de crianças pequenas implantadas as versões no português brasileiro da *Infant Toddler Meaningful Auditory Integration Scale* (IT-MAIS)^(13)^, do Questionário Auditivo *LittlEars®* ^(14)^ e da *Meaningful Use of Speech Scale* (MUSS)^(15)^, dentre outros. Os dois primeiros avaliam a percepção auditiva da fala, enquanto o MUSS avalia o uso da linguagem falada. No entanto, esses instrumentos foram padronizados para crianças sem deficiências adicionais.

Partindo do pressuposto de que é comum que crianças surdas com múltiplas deficiências apresentem um desenvolvimento comunicativo mais lento após o implante coclear^(2-4)^, a elaboração de instrumentos direcionados a essa população torna-se fundamental.

Até o momento, não existem pesquisas realizadas no Brasil com uso do DAD-Q, já que o instrumento estava disponível somente na versão em inglês^9^. A tradução e adaptação cultural do questionário foi realizada com o objetivo de preencher uma lacuna na avaliação do benefício do implante coclear em crianças surdas com deficiências adicionais. O questionário não se limita a avaliação da percepção auditiva e linguagem falada, mas sim considera o desempenho comunicativo da criança, incluindo questões sobre atenção, interação e método preferencial de comunicação. Isso é importante ao avaliar crianças com deficiências adicionais, que muitas vezes podem não apresentar o benefício esperado do implante coclear para a comunicação oral, porém podem demonstrá-lo em outras habilidades comunicativas, como na interação^(16)^. Além disso, considerando que é essencial que o profissional realize o aconselhamento a respeito das expectativas dos pais sobre o uso do implante coclear^(5)^, o uso do DADQ-PT para avaliação de crianças surdas com diferentes deficiências adicionais pode ajudar a fornecer informações sobre o que esperar do uso do dispositivo nessas populações, contribuindo para direcionar as orientações durante o aconselhamento.

Sabe-se que a validação de instrumentos após a tradução é um processo contínuo. Sendo assim, sugere-se que futuras pesquisas sejam realizadas por meio do uso do DADQ-PT em amostras maiores e variadas para sua normatização no país.

## CONCLUSÃO

O instrumento *Deafness and Additional Disabilities Questionnaire* (DAD-Q) foi traduzido e adaptado culturalmente para o português brasileiro, resultando no “Questionário de Surdez e Deficiências Adicionais (DADQ-PT)”, tornando possível disponibilizar para os profissionais da área de (re)habilitação auditiva um instrumento inédito e útil para avaliação do benefício do implante coclear em crianças com perda auditiva e deficiências adicionais.

## Apêndice A Questionário de Surdez e Deficiências Adicionais (DADQ-PT)

**Table:**

**Questionário de Surdez e Deficiências Adicionais (DADQ-PT)**									
**Avaliação do desenvolvimento comportamental, auditivo, comunicativo, atencional e social, pré e pós-implante coclear**									
**PESQUISA PARENTAL**									
**Nome__________________________ Preenchido por______________________ Data____________**									
**⬜ Pré-IC ⬜ Pós-IC**									
**Aplicação:** este questionário deve ser aplicado em formato de entrevista. As perguntas foram elaboradas para elucidar um diálogo entre examinador e informante. Fazer com que os próprios informantes (ex.: pais) preencham o instrumento, invalida o procedimento.									
**Instruções para o examinador:** pergunte ao informante: “Gostaríamos de saber sobre suas impressões a respeito do desenvolvimento comportamental, auditivo e comunicativo de seu filho. A cada pergunta, tente se lembrar e me contar o máximo de exemplos possíveis sobre esse comportamento e depois tente definir com que frequência a criança demonstra esse comportamento específico, de forma sistemática.”									
**Resposta**			**Abreviatura**	**Escore**
**Nunca**			N	0
**Raramente**			R	1
**Algumas vezes**			AV	2
**Muitas Vezes**			MV	3
**Sempre**			S	4
**A) Habilidades Perceptivas**									
1		Por quanto tempo a criança usa o implante coclear durante as horas em que está acordado?					N R AV MV S		
		(N: [0%]; R: [1%-25%]; AV [25%-50%]; MV [50%-75%]; S [>75%])							
2		Liga o processador de fala sozinho ou pede a alguém que o faça (ex.: aponta para o dispositivo, mesmo sem verbalização)?					N R AV MV S		
3		Percebe quando o dispositivo não está funcionando adequadamente (ex.: qualidade/volume)?					N R AV MV S		
4		Atende ao próprio nome quando chamado inesperadamente em um local silencioso (sem nenhuma pista visual)?					N R AV MV S		
5		Atende ao próprio nome quando chamado em um local ruidoso?					N R AV MV S		
6		Detecta o barulho de eletrodomésticos?					N R AV MV S		
7		Reage a sons em ambiente domiciliar (telefone tocando, batidas à porta, campainha etc.)?					N R AV MV S		
8		Reage aos sons de animais (miado, latido etc.)?					N R AV MV S		
9		Reconhece um animal pelo som que ele faz (ex: associa latido a um cachorro)?					N R AV MV S		
10		Percebe quando alguém está falando mesmo sem estar vendo a pessoa?					N R AV MV S		
**B) Modo preferencial de comunicação**									
Qual é o modo preferido utilizado pela criança para se comunicar?									
(0) Comportamento (ex. sorrisos, gritos, expressões faciais)									
(1) Gestos									
(2) Gestos e vocalizações									
(3) LIBRAS ou CSA (Comunicação Suplementar e Alternativa)									
(4) Gestos associados a sílabas									
(5) Palavras associadas a gestos									
(6) Palavras isoladas									
(7) Frases simples (2-3 palavras)									
(8) Sentenças completas									
**C) Comportamentos comunicativos**									
1		Tenta se comunicar com conhecidos, ex.: familiares, amigos, professores etc. (utilizando qualquer modo de comunicação)?					N R AV MV S		
2		Tenta se comunicar com desconhecidos (utilizando qualquer modo de comunicação)?					N R AV MV S		
3		Respeita as trocas de turnos na conversação?					N R AV MV S		
4		Sua fala é inteligível para seus familiares?					N R AV MV S		
5		Sua fala é inteligível para desconhecidos?					N R AV MV S		
6		Tenta se corrigir se as pessoas não o(a) entendem (utilizando qualquer modo de comunicação)?					N R AV MV S		
7		Consegue se comunicar ao telefone?					N R AV MV S		
8		Utiliza a fala como o modo de comunicação preferido para chamar alguém?					N R AV MV S		
9		Consegue comunicar suas necessidades (utilizando qualquer modo de comunicação)?					N R AV MV S		
**D) Atenção e habilidades de memória**									
1		É capaz de focar a atenção no ambiente?					N R AV MV S		
2		É capaz de focar a atenção em uma tarefa por tempo suficiente para executá-la?					N R AV MV S		
3		Demonstra escutar o que está sendo dito ou comunicado a ele(a)?					N R AV MV S		
4		Evita comportamentos como correr e subir nos lugares ou contorcer-se em sua cadeira ou remexer suas mãos ou pés em momentos inadequados?					N R AV MV S		
5		É capaz de manter sua atenção em uma tarefa e realizá-la, mesmo na presença de outros estímulos?					N R AV MV S		
6		Parece se lembrar de pessoas conhecidas (ex: parentes, professores, terapeutas)?					N R AV MV S		
7		É capaz de se lembrar de palavras conhecidas?					N R AV MV S		
8		É capaz de se lembrar das regras de um jogo simples?					N R AV MV S		
**E) Interação social, controle comportamental e autorregulação**									
1		Acena espontaneamente ao encontrar pessoas conhecidas?					N R AV MV S		
2		Interage adequadamente com outros (chama sua atenção; mantém distância recíproca; respeita os turnos da conversação)					N R AV MV S		
3		Gosta de ir à escola?					N R AV MV S		
4		Interage com familiares de maneira apropriada?					N R AV MV S		
5		Interage com os colegas de classe de maneira apropriada?					N R AV MV S		
6		Gosta de fazer as sessões de reabilitação?					N R AV MV S		
7		Age de forma independente durante as atividades de vida diária (ex.: tomar banho, comer etc.)? No caso de criança muito pequena ou com alterações motoras: parece desejar agir de forma independente?					N R AV MV S		
8		Demonstra ampla gama de emoções e afetos (ex.: feliz ao brincar com os colegas, concentrado ao fazer exercícios, triste ao ser castigado?)					N R AV MV S		
9		Fica feliz por estar na companhia de alguém?					N R AV MV S		
10		Entende piadas?					N R AV MV S		
11		Inicia uma atividade espontaneamente (ex.: escolhe e propõe jogos por conta própria)?					N R AV MV S		
12		Compreende quando é repreendido?					N R AV MV S		
13		Gosta de assistir TV?					N R AV MV S		
14		Gosta de escutar música?					N R AV MV S		
**Perguntas adicionais (somente para avaliação pós-IC)**									
1		Você acha que recebeu informações suficientes do centro de implante coclear na época para tomar a decisão correta?						SIM NÃO	
2		Você conheceu outras famílias de crianças implantadas com outras deficiências adicionais antes do implante de seu(sua) filho (a), cuja experiência o(a) ajudou a tomar sua decisão?						SIM NÃO	
3		Você sugeriria o implante coclear para um outro pai que tenha um filho com condição semelhante?						SIM NÃO	
4		Sua família concordou com a sua decisão?						SIM NÃO	
5		A criança foi diagnosticada com deficiências adicionais antes do implante coclear?						SIM NÃO	
6		Se você precisasse tomar a decisão sobre um implante coclear para seu (sua) filho(a) novamente, você optaria pelo implante coclear?						SIM NÃO	
**Escores**								
**A**	**Habilidades perceptivas**							
**B**	**Modo preferencial de comunicação**							
**C**	**Comportamentos comunicativos**							
**D**	**Atenção e habilidades de memória**							
**E**	**Interação social, controle comportamental e autorregulação**							
**Escore total**
